# Supplementary figures and images for: Extraction and high-throughput sequencing of oak heartwood DNA: Assessing the feasibility of genome-wide DNA methylation profiling
Source: PLoS One. 2021 Nov 18;16(11):e0254971. doi: 10.1371/journal.pone.0254971 (PMC8601515; doi:10.1371/journal.pone.0254971)

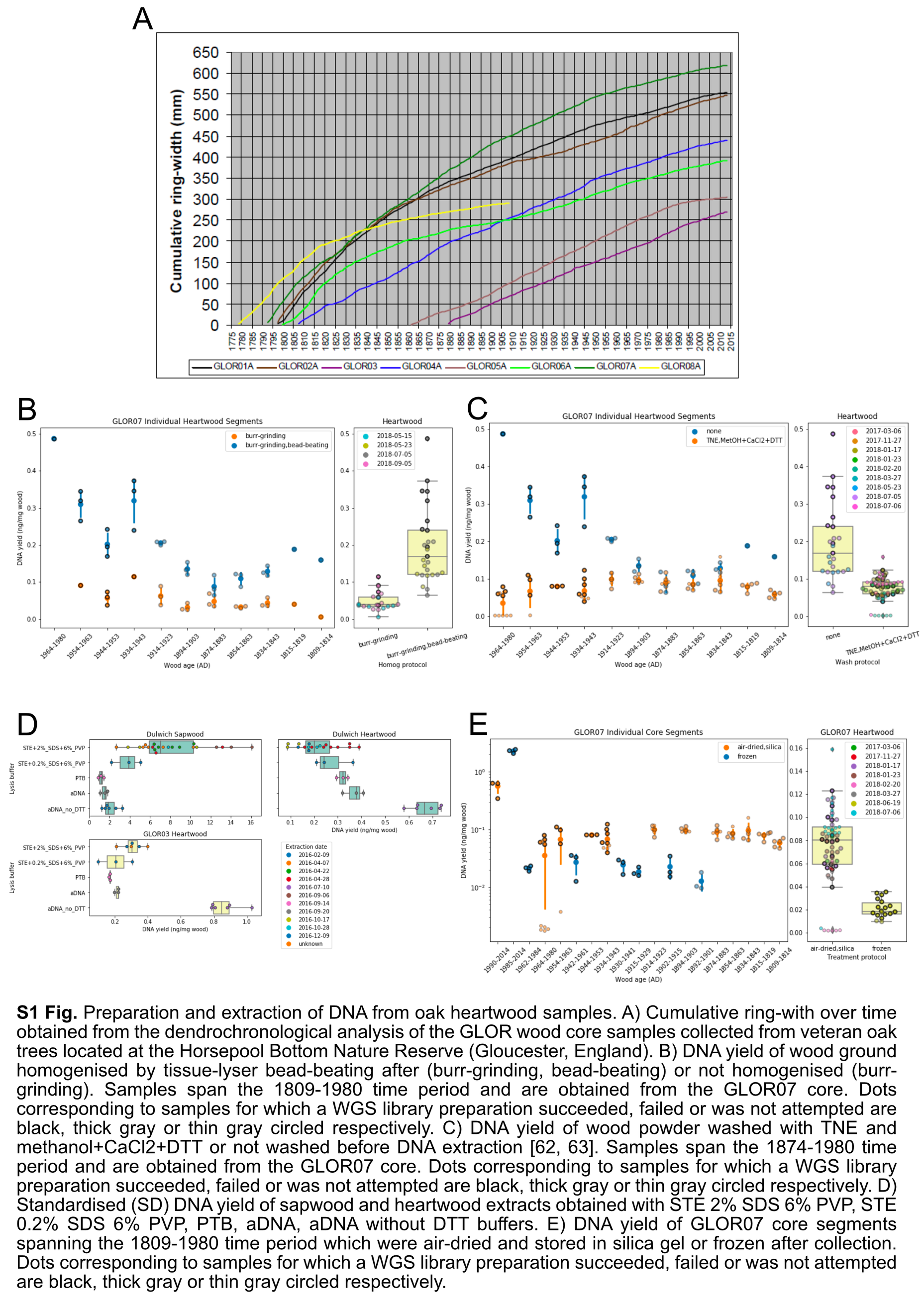

Supplement: S1 Fig — A) Cumulative ring-with over time obtained from the dendrochronological analysis of the GLOR wood core samples collected from veteran oak trees located at the Horsepool Bottom Nature Reserve (Gloucester, England). B) DNA yield of wood ground homogenised by tissue-lyser bead-beating after (burr-grinding, bead-beating) or not homogenised (burr-grinding). Samples span the 1809-1980 time period and are obtained from the GLOR07 core. Dots corresponding to samples for which a WGS library preparation succeeded, failed or was not attempted are black, thick gray or thin gray circled respectively. C) DNA yield of wood powder washed with TNE and methanol+CaCl2+DTT or not washed before DNA extraction [62, 63]. Samples span the 1874-1980 time period and are obtained from the GLOR07 core. Dots corresponding to samples for which a WGS library preparation succeeded, failed or was not attempted are black, thick gray or thin gray circled respectively. D) Standardised (SD) DNA yield of sapwood and heartwood extracts obtained with STE 2% SDS 6% PVP, STE 0.2% SDS 6% PVP, PTB, aDNA, aDNA without DTT buffers. E) DNA yield of GLOR07 core segments spanning the 1809-1980 time period which were air-dried and stored in silica gel or frozen after collection. Dots corresponding to samples for which a WGS library preparation succeeded, failed or was not attempted are black, thick gray or thin gray circled respectively. (TIF) [file pone.0254971.s001.tif]
