# Supplementary material for: Extraction and high-throughput sequencing of oak heartwood DNA: Assessing the feasibility of genome-wide DNA methylation profiling
Source: PLoS One. 2021 Nov 18;16(11):e0254971. doi: 10.1371/journal.pone.0254971 (PMC8601515; doi:10.1371/journal.pone.0254971)
Supplement: S1 Raw images — (PDF) [file pone.0254971.s006.pdf]

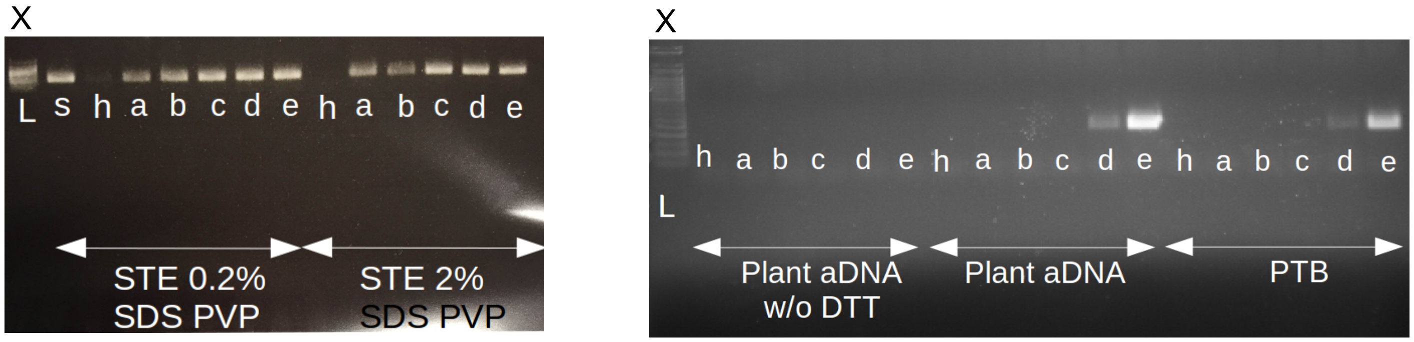

Figure 1A) PCR inhibitory effect of heartwood DNA extracts obtained with STE 2% SDS 6% PVP, STE 0.2% SDS 6% PVP, aDNA buffer with and without DTT, PTB buffer. Amplifiable sapwood DNA extract (s) was titrated with heartwood DNA extract (h) at 1:4 (a), 2:4 (b), 3:4 (c), 3.25:4 (d), 3.5:4 (e). Sapwood heartwood extract DNA was amplified by PCR for 40 cycles. The two panels of this figure were taken from different gels under the same experimental conditions. The letter "X" was reported above the lanes containing the 50 bp ladders (L) as those were not included in the figure reported in the main text. Images were obtained through a gel imaging system of the gel after the completion of the electrophoresis.

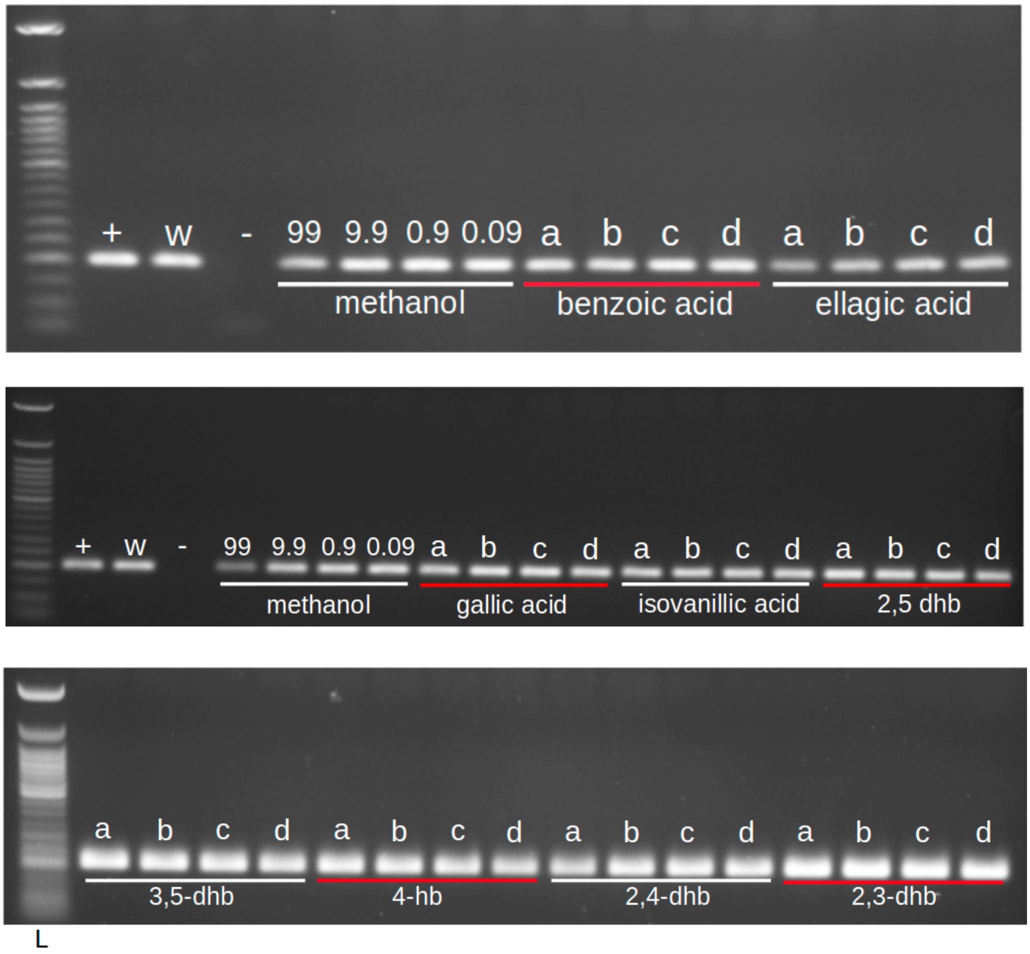

Figure 1D) Concentration of the tested compound: a) 1.0 mM, b) 0.1 mM, c) 0.01 mM, d) 0.001 mM. Sapwood (+), 50% diluted sapwood (w), PCR negative control (-). Hb: hydroxybenzoic acid, dhb: dihydroxybenzoic acid. Ellagic acid is dissolved in methanol which is why controls with the addition of only methanol to the PCR were carried out (99%, 9.9%, 0.9%, 0.09%). The 50 bp ladder was contained in the lane indicated with the letter "L". The upper, middle, and lower panels of this figure were taken from different gels under the same experimental conditions. Images were obtained through a gel imaging system of the gel after the completion of the electrophoresis.
