# Supplementary material for: Extraction and high-throughput sequencing of oak heartwood DNA: Assessing the feasibility of genome-wide DNA methylation profiling
Source: PLoS One. 2021 Nov 18;16(11):e0254971. doi: 10.1371/journal.pone.0254971 (PMC8601515; doi:10.1371/journal.pone.0254971)
Supplement: Attachment — Submitted filename: rebuttal_final.pdf [file pone.0254971.s007.pdf]

King's College London
Faculty of Life Sciences & Medicine
School of Basic and Biomedical Sciences

Head of School
Professor Mathias Gautel

Head of Department
Professor Tim J P Hubbard

Department of Medical & Molecular Genetics
Floor 8 Tower Wing
Guy's Hospital
London SE1 9RT

KING'S College LONDON
University of London

The Academic Editor

Dr Emidio Albertini

PLoS ONE

Please respond to:

Reiner Schulz, PhD
Visiting Senior Lecturer in Bioinformatics & Epigenomics
Email: reiner.schulz@kcl.ac.uk

27th September 2021

Dear Dr. Albertini,

Thank you for the opportunity to revise our submission. Below, please find our responses to your and the reviewers' comments.

Academic Editor's comments

1. We have reviewed the style requirements. We have found the following deviations and rectified them: The supplementary tables 1 and 2 that were referred to in the text as "Table S1 and Table S2" are now referred to as "S1 and S2 Tables". Moreover, tables that were cited in the text through the abbreviation "Tab." are now cited as "Table". The format of the tables and of the author affiliations was changed to meet the requirements of the journal. Please note that in addition, we in the authors list, we corrected the affiliation index for Laura Southgate to be 1 instead of 8.
2. We have added a new table (Table 1) containing the girth, National Grid References and species for each of the trees. This also addresses comment 3 of reviewer 1 (see below).
3. We have added the following statement to the first paragraph of the M&Ms subsection "Wood core sampling and preparation": "*No sampling permits were required. The Horsepool Bottom Nature Reserve is privately owned and operated by co-author Dr Andy Moir, who also performed the core sampling. The Dulwich Woods sample was a discarded trunk cross-section, left on the ground after public footpath maintenance.*"
4. We have reworded the Acknowledgements section to read: "*We thank Fabrizio Cleri (University of Lille, France) for useful discussions on DNA modelling.*"
   The Funding Statement is correct as is and need not be changed.
5. The accessions (GEO GSE143201 and DOI 10.18742/RDM01-502) are stated in the Data Availability section.
6. A single PDF file named S1_raw_images with the uncropped gel images has been uploaded as a Supporting Information file.
7. Based on an email exchange with Daiby Santos, Straive Editorial Assistant, we understand that panels A, B, D, G, and H of Figure 2 (previously Figure 1) were flagged as similar to copyrighted

material. All of these panels contain molecular structure visualisations that have been *de novo* rendered by the Visual Molecular Dynamics software package (https://www.ks.uiuc.edu/Research/vmd/allversions/what_is_vmd.html). As such, they are original work, for which the manuscript authors have the copyright.

8. Tables 1 and 2 were referred to in the main text (lines 57, 59 and 72) as Tab. 1 and Tab. 2. We have changed the main text references to read “Table” instead of “Tab.”. Supplementary tables S1, S2 and S3 in Supplementary_Tables.pdf were referred to in the main text (lines 88, 94, 181, 218, 222, 339, 344, 371, 373, 386 and 461) as S1 Table, S2 Table and S3 Table. We have modified the references to the Supplementary Tables to adhere to the style requirements, that is, for example, “S1 and S2 Tables” instead of “S1 Table and S2 Table”.

Please note that in addition, we removed the primer sequences for the *FT2* and *LFY* genes from supplementary table 2 since they were not used in the manuscript.

Reviewer #1 comments

1. We thank the reviewer for their interest and answer their question(s) below. We have not made any changes to the manuscript in response to this comment.
   In the exploratory phase of the project, we investigated many DNA extraction options, including kits whose manufacturers claim they have been optimised for application to phenolic compound-rich plant material, e.g., kits by Qiagen (Plant mini), MoBio (Powerplant Pro) and Analytik Jena (innuPrep Plant DNA). We found that none of the kits performed qualitatively better than the non-kit, SDS-based extraction protocol we present. We speculate that this is because heartwood is qualitatively different from the difficult plant materials for which the kits have been optimised. We did not find that a larger amount of heartwood sample compensated for DNA fragmentation. We tested this by pooling the DNA extracted from multiple heartwood samples. Given the small amounts of DNA contained in heartwood, gel-based selection of higher molecular weight DNA was not an option, and whole genome amplification prior to size selection would have removed the DNA methylation. It may be possible to use microfluidics to select for higher molecular weight DNA and to then pool samples to obtain a sufficient amount for sequencing library preparation. However, that was beyond the budget of the project. The pulverizer we used can process 12 samples in parallel, and there are other, more expensive models that can process 96 samples. However, the drilled cores have a diameter of 12mm so that at an annual growth of 1mm and oak wood density of 0.5mg/mm^3, a core segment encompassing 5 years of growth weighs 250mg. Therefore, pulverizing very large amounts/long core segments of heartwood implies losing temporal resolution.
2. We thank the reviewer for their interest and answer their question(s) below. We have not made any changes to the manuscript in response to this comment.
   We used the Kaiju tool (https://kaiju.binf.ku.dk/) by Menzel et al. (Nature Commun. 7:11257, 2016) to taxonomically classify heartwood reads that did not align to the oak reference genome. Between 2% and 22% of unaligned reads could be classified. In most cases, bacterial and fungal DNA dominated (>50% and up to 90%).
3. The trees sampled at the Horsepool Bottom Nature Reserve are of the *Quercus robur* species. The Dulwich Woods sample was a cross-section of a recently felled tree belonging to a stand comprising specimen of the closely related and often hybridizing species *Q. robur* and *Q. petreae*.

We have modified the manuscript in the M&M subsection "Wood core sampling and preparation" to read: "[...] four oak (*Quercus robur*) trees [...]" (line 55), and "[...] a core cross-section of an oak (*Quercus robur* or *Quercus petraea*) tree trunk collected in the Dulwich Woods, NGR: TQ 34340 72608 (5 Peckarmans Wood, London SE26 6SB, United Kingdom) (Table 1)." (line 57). We also replaced two occurrences of "Dulwich Wood" with the correct "Dulwich Woods" place name.

Reviewer #2 comments

We thank reviewer #2 for their eagle-eyed, constructive comments.

Line 82: We have added the assay name. The text now reads: *"[...] (Qubit 3.0 Fluorometer, dsDNA HS assay, ThermoFisher Scientific)."* (line 88)

Line 83: We have added an additional table to the Supplementary Materials, which, for all 427 DNA-extracted samples, contains the measured DNA concentrations as well as the measured phenolic compound concentrations where available and all the available experimental meta-data. We reference this table in the text, which now reads: *"[...] DNA was extracted from 427 samples obtained from 7 distinct oak wood cores/cross-section (S1 File)."* (line 89)

Lines 170-186: The referenced protocol by Meyer and Kircher details all enzymes, including manufacturers and catalog numbers. However, we sincerely thank the reviewer for flagging the uracil issue, which required the use of a different polymerase, which we now specify in the text: *"Indexing-PCR was performed for 24 cycles for both WGS and WGBS libraries with a proofreading but uracil-tolerant DNA polymerase (Phusion U Hot Start DNA polymerase, Thermo Scientific, cat. no. F555S), and adapter-dimers were removed as for WGS libraries [49]."* (line 184)

Figures 3D, E, F: We disagree on this point since box/violin plots would not allow the visualization and comparison of the qualitatively different fitted logistic regression models and their confidence intervals (CIs). The visual contrast between Figures 3D&E (barely sigmoidal with wide CIs) and Figure 3F (sigmoidal with narrow CIs) illustrates that DNA or phenolic compound concentration alone are not predictive of library preparation success, while the DNA concentration residuals are. We would therefore prefer to keep these figures as they are.

Figures 5B, C & D: For brevity, the original panel A of Figure 5 was removed prior to submission, but the legend was not updated. The original part A of the legend has been removed, and the other panel references have been changed accordingly (B->A, C->B, D->C).

Figure 2 first, then Figure 1: We agree with the reviewer. The main text presents the results illustrated by Figure 2 first, followed by the results illustrated by Figure 1. We consequently swapped the order of the figures to correspond with the order of the results presentation in the main text.

Size distribution of the extracted DNA: We agree with the reviewer that it would be useful to know the size distribution of extracted heartwood DNA. We attempted to directly determine it using microfluidics (Agilent Bioanalyzer; HS DNA assay with up to 10kbp size range), but the assay consistently failed, presumably due to the presence of Bioanalyzer -incompatible impurities in the extracts. However, our lack of success amplifying the 203 bp region of the chloroplast from heartwood DNA suggests that no HMW DNA was present, consistent with the reviewer's view that the presence of

HMW DNA is unlikely. We hypothesized that fragmentation was to blame and therefore performed library preparations w/o a fragmentation step since that would only have exacerbated the problem. Because we did not include a fragmentation step, the fragment length distribution derived from the sequencing results ought to be representative of the input DNA, and, as opposed to what we see with sapwood, the distribution does not have a long tail, again suggesting an absence of HMW DNA in heartwood rather than its non-incorporation into the library. We also note that the Phusion U Hot Start DNA Polymerase that we used for DNA library amplification, according to the manufacturer, can generate amplicons up to 20kbp, but we did not see any mapped fragments even approaching that size. We have not changed the manuscript in response to this comment.

***Pre*- versus *post*-BS library preparation**: We indeed cannot repeat the experiments using a different library preparation method. At the time, we decided to adapt the Meyer & Kircher (M&K) WGS library preparation method for WGBS (methylated adapters, BS treatment *post* the addition of adapters) since M&K worked in our hands down to 50pg of input DNA and has been used for ancient DNA studies. It is possible that a different library preparation method would work better for the purpose of whole genome DNA methylation profiling of heartwood DNA. The Conclusion section already contained a statement and reference of a BS-free method of direct methylation sequencing. We have added a discussion of the post-bisulfite adapter tagging (PBAT) method as a promising alternative to the same paragraph of the Conclusion.

Sincerely yours,

Reiner Schulz, PhD
